# GaitScreen: a ground reaction force-based framework for prescreening gait abnormalities in orthopaedic populations

**DOI:** 10.3389/fbioe.2026.1947887

**Published:** 2026-10-26

**Authors:** Qingyao Bian, Qing Zhang, Yang Li, Ziyun Ding

**Affiliations:** 1 School of Engineering, University of Birmingham, Birmingham, United Kingdom; 2 Gait and Motion Analysis Center, Yueyang Hospital of Integrative Medicine, Shanghai University of Traditional Chinese Medicine, Shanghai, China

**Keywords:** decision support, gait prescreening, ground reaction force, pathological gait, rehabilitation triage

## Abstract

Ground reaction force (GRF) trajectories capture kinetic alterations in stance-phase loading, propulsion, and mediolateral control. This study presents GaitScreen, a GRF-based framework for post-acquisition prioritisation of already recorded healthy-control and pathological-gait trials. GaitScreen converts vertical, anterior–posterior, and mediolateral stance-phase GRF trajectories into a review-prioritisation score and a threshold-dependent review flag. The framework combines a multiscale temporal backbone, a convolutional neural network component, and a logistic regression component to integrate multiscale force patterns, learned convolutional representations, and raw-waveform linear information. It was evaluated using the GaitRec dataset, comprising healthy controls and individuals with lower-limb orthopaedic conditions. Under a subject-exclusive evaluation protocol, GaitScreen achieved consistent trial-level performance across ten matched runs, with an accuracy of 0.8766±0.0025 , a macro-F1 score of 0.8757±0.0024 , and an AUROC of 0.9425±0.0015 . A high-sensitivity operating mode reduced the false-negative rate from 0.1736 to 0.1073, with an increase in false-positive review flags. The full hybrid model had a serialized size of 1.122 MB and a mean CPU inference latency of 0.626±0.010 ms per sample after GRF preprocessing. The compact model size and low inference latency support computationally scalable post-acquisition processing of standardised GRF trials.

## 1 Introduction

Gait is an information-rich functional activity that reflects the integrated performance of the nervous, musculoskeletal, sensory, cognitive, and cardiorespiratory systems (Pirker and Katzenschlager, 2017; Studenski et al., 2011). Alterations in walking speed, rhythm, symmetry, and loading behaviour can indicate impaired mobility, balance control, functional capacity, and compensatory movement strategies (Whittle, 1996; Baker, 2006; Simon, 2004; Pirker and Katzenschlager, 2017). Automated analysis of these characteristics can support the review of gait recordings from neurological and musculoskeletal populations (Astephen et al., 2008; Hodges and Tucker, 2011; Perry and Burnfield, 2010).

Objective gait analysis can draw on spatiotemporal characteristics (Viteckova et al., 2020; Ghoraani et al., 2021), kinematic patterns (Whittle, 1996; Baker, 2006), kinetic signals (Simon, 2004; Baker, 2006), and muscle activation measurements (Winter 1991; Perry and Burnfield, 2010). Among these, ground reaction force (GRF) trajectories provide direct kinetic information on weight acceptance, body support, braking, propulsion, and mediolateral force regulation during walking (Winter 1991; Baker, 2006; Simon, 2004). These characteristics may be altered in pathological gait (Shuzan et al., 2023; Farashi, 2020; Slijepcevic et al., 2018), making GRF a biomechanically meaningful signal for automated analysis when standardised force recordings are available.

Several studies have investigated GRF-based discrimination between healthy and pathological gait. Slijepcevic et al. evaluated GRF parameters and PCA-based representations with classical classifiers for binary and multiclass functional-gait classification (Slijepcevic et al., 2018). Subsequent GaitRec studies introduced a one-dimensional convolutional network for binary gait-disorder detection (Pandey et al., 2022), boosted classifiers for detailed abnormality classification (Jani et al., 2022), and an explainable-learning pipeline combining supervised classifiers with GMM-LIME explanations (Mulwa et al., 2024). Wang et al. more recently applied a Transformer-style temporal model to five-class classification of healthy gait and lower-limb impairment groups (Wang et al., 2025). Collectively, these studies show that GRF representations and multiple model families have been used for binary and multiclass gait classification.

Methodological differences in input representation, class definition, model family, and evaluation design limit direct comparison across prior studies. To provide a common basis for analysis, the present study evaluates all models under one subject-exclusive protocol. The protocol combines validation-selected operating thresholds with sensitivity, specificity, false-negative rate, and false-positive rate at the selected operating points. Computational compactness, component and fusion behaviour, and the effects of *post hoc* subject aggregation and calibration are examined within the same framework.

This study makes four contributions. First, it develops a compact hybrid model that integrates multi-scale temporal, conventional convolutional, and 303-dimensional raw-waveform linear representations. Second, it applies a common subject-exclusive comparison protocol with validation-selected operating thresholds and ten matched-seed refits under the same fixed partition. Third, it characterises threshold-dependent sensitivity, specificity, false-negative and false-positive behaviour, pathology-family variation, and computational cost for post-acquisition trial review. Fourth, it examines the model through temporal-scale and component-fusion analyses, backbone-level feature attribution, and *post hoc* subject-level aggregation and calibration.

## 2 Materials and methods

### 2.1 Problem statement

The intended use is post-acquisition prioritisation of already recorded gait trials for further review. The screening formulation is defined in Equations 1–4. We formulate this task as a thresholded review-prioritisation problem. Let

$$D={\left\{\left(x_{i},y_{i}\right)\right\}}_{i=1}^{N}$$ (1)

denote the gait dataset, where $x_{i}\in R^{C\times T}$ represents a limb-specific ground reaction force (GRF) sequence and $y_{i}\in \left\{0,1\right\}$ denotes the reference screening status.

The primary GaitScreen model does not rely on a predefined set of handcrafted biomechanical GRF parameters. Each input trial comprises the complete time-normalised vertical, anterior–posterior, and mediolateral GRF trajectories, with 101 stance-phase samples per channel; thus, C=3 , T=101 , and each trial contains 3×101=303 waveform values. These three components were defined as model inputs before training and before the interpretability analysis. Vertical GRF primarily represents body support and stance-phase loading, anterior–posterior GRF captures braking and propulsion, and mediolateral GRF characterises side-to-side force regulation; together, they provide complementary directional information about stance-phase interaction with the ground (Winter 1991; Baker, 2006; Simon, 2004). In GaitRec, GRF amplitudes are expressed as multiples of body weight, and directional signs are standardised such that anterior and medial forces are positive (Horsak et al., 2020). The multi-scale temporal and CNN branches retain the channel-by-time structure, whereas the raw-waveform Logistic Regression branch uses the same 303 values after flattening.

The reference screening status is defined as

$$y_{i}=\left\{\begin{array}{cc} 0, & \text{healthy}-\text{control gait},\\ 1, & \text{pathological}-\text{gait}. \end{array}\right.$$ (2)

The model output is a review-prioritisation score,

$$s_{i}=f\left(x_{i}\right)\in \left[0,1\right],$$ (3)

expressed on a probability scale. A validation-selected threshold τ converts this score into a review flag,

$${\hat{r}}_{i}\left(\tau \right)=\left\{\begin{array}{cc} 1, & s_{i}\geq \tau ,\\ 0, & s_{i}<\tau . \end{array}\right.$$ (4)

A positive flag indicates that the trial is prioritised for further clinical review.

Under this formulation, false-negative flags correspond to pathological-gait trials not prioritised for review, whereas false-positive flags correspond to healthy-control trials prioritised for review. The evaluation therefore reports sensitivity, specificity, false-negative rate, false-positive rate, and threshold-dependent review behaviour.

### 2.2 Dataset and preprocessing

Experiments were conducted using the public GaitRec dataset (Horsak et al., 2020), a large benchmark of standardised bilateral stance-phase GRF recordings from healthy-control participants and individuals with lower-limb musculoskeletal gait impairments. The original dataset includes 211 healthy-control participants and 2,084 patients, with more than 75,000 bilateral GRF trials acquired under a standardised walking protocol. The pathological cohort is organised into hip, knee, ankle, and calcaneus impairment families, with detailed subclass labels available for each family. The following preprocessing steps were applied to construct the retained analysis cohort and support subject-exclusive evaluation.

GaitRec was selected because it combines a comparatively large public cohort, standardised three-component force-plate GRF recordings, subject identifiers, and lower-limb orthopaedic metadata suitable for subject-exclusive evaluation. Other public gait datasets differ in population, sensing modality, and label structure and are therefore not included in this study.

Accordingly, retained trials were restricted to self-selected walking speed and normal-shoe conditions; both limbs were retained for healthy-control participants, whereas the clinically affected limb or limbs were retained for pathological participants. Only the first eligible session for each subject was used, with up to five stance-phase trials from each retained limb, and detailed subclasses that could not support subject-exclusive allocation were excluded, leaving 23 retained pathological subclasses. The final limb-level screening cohort comprised 2,189 subjects, including 208 healthy-control participants and 1,981 pathological participants, yielding 12,089 limb-specific GRF trials, with 2,078 healthy-control trials and 10,011 pathological-gait trials. No additional temporal resampling, handcrafted feature extraction, or centre-of-pressure information was used.

Instead of adopting the official GaitRec train–test split, retained subjects were allocated once to a subject-exclusive train/validation/test partition after fine stratification across detailed pathology subclasses, which preserved pathology-family composition before defining the final healthy-control-versus-pathological-gait screening reference status. The held-out test set comprised 1,228 limb-specific trials, including 640 healthy-control trials and 588 pathological-gait trials; healthy-control trials were oversampled with replacement only within the training subset, with no oversampling applied to validation or test sets. Detailed preprocessing and cohort construction procedures are provided in Supplementary Method S1.

This study was a secondary analysis of the publicly available, completely anonymised GaitRec dataset. Approval for the original data collection and dataset publication was granted by the Ethics Committee of Lower Austria (GS1-EK-4/299–2014) (Horsak et al., 2020). No new participants were recruited and no identifiable data were accessed.

### 2.3 GaitScreen

GaitScreen converts recorded stance-phase GRF trials into review-prioritisation scores and threshold-dependent review flags, as illustrated in Figure 1. Given an input trial $x\in R^{3\times 101}$ , GaitScreen estimates the score by combining probability estimates from a multi-scale temporal backbone branch, a conventional CNN branch, and a raw-waveform Logistic Regression branch. The model components and hybrid scoring rule are defined in Equations 5–19, while detailed architectural settings and hybrid-inference specifications are provided in Supplementary Method S2.

**FIGURE 1:**
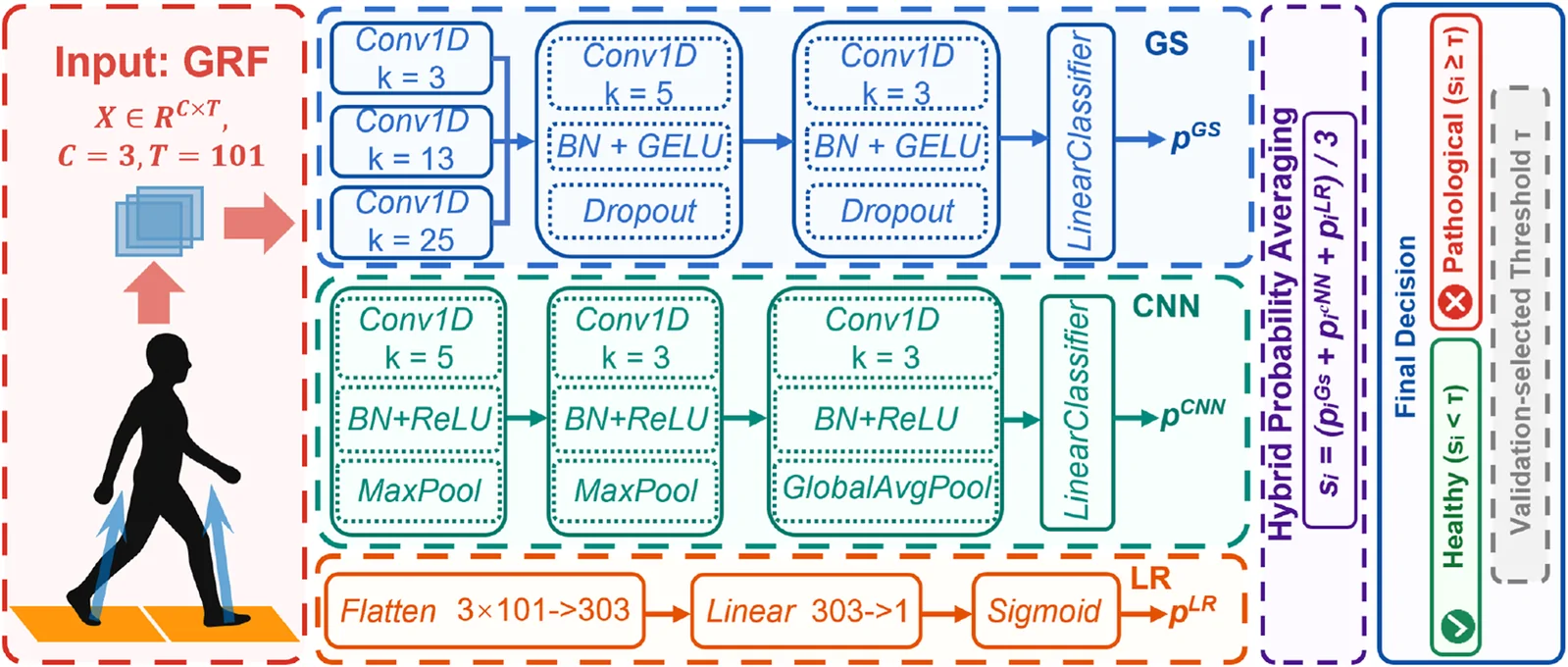
Overall GaitScreen workflow for post-acquisition trial review prioritisation. A recorded stance-phase GRF trial is processed by a multi-scale temporal backbone, a CNN component, and a raw-waveform Logistic Regression component. Their pathological-class probabilities are averaged to produce the GaitScreen score, which a validation-selected threshold converts into a review flag.

#### 2.3.1 Multi-scale temporal backbone branch

The multi-scale temporal backbone branch extracts local and medium-range stance-phase GRF patterns. For compact notation, let

$$E_{q}\left(a\right)=Dropout\;\left(GELU\;\left(BN\;\left({Conv1D}_{q}\left(a\right)\right)\right)\right).$$ (5)

The input trial is first processed by three parallel convolutional stems and concatenated channel-wise.

$$h^{\left(k\right)}={Conv1D}_{k}\left(x\right),\;k\in \left\{3,13,25\right\},$$ (6)

$$h^{\text{ms}}=Concat\;\left(h^{\left(3\right)},h^{\left(13\right)},h^{\left(25\right)}\right).$$ (7)

The concatenated representation is refined by two lightweight convolutional encoding blocks.

$$u^{\left(1\right)}=E_{5}\;\left(h^{\text{ms}}\right),$$ (8)

$$u^{\left(2\right)}=E_{3}\;\left(u^{\left(1\right)}\right).$$ (9)

A linear classifier maps the encoded representation to the pathological-class probability:

$$p^{\text{GS}}={\left[Softmax\;\left(W_{\text{GS}}\phi \left(u^{\left(2\right)}\right)+b_{\text{GS}}\right)\right]}_{1},$$ (10)

where ϕ(⋅) denotes the flattening or pooling operation before the classifier. This branch captures both short-range loading transients and broader stance-phase force-evolution patterns while remaining computationally compact.

#### 2.3.2 CNN branch

The CNN branch provides a conventional convolutional representation of the same GRF trial. For compact notation, let

$$C_{q}\left(a\right)=ReLU\;\left(BN\;\left({Conv1D}_{q}\left(a\right)\right)\right).$$ (11)

The three CNN blocks are defined as.

$$c^{\left(1\right)}=MaxPool\;\left(C_{5}\left(x\right)\right),$$ (12)

$$c^{\left(2\right)}=MaxPool\;\left(C_{3}\left(c^{\left(1\right)}\right)\right),$$ (13)

$$c^{\left(3\right)}=GlobalAvgPool\;\left(C_{3}\left(c^{\left(2\right)}\right)\right).$$ (14)

The CNN pathological-class probability is obtained as

$$p^{\text{CNN}}={\left[Softmax\;\left(W_{\text{CNN}}c^{\left(3\right)}+b_{\text{CNN}}\right)\right]}_{1}.$$ (15)

#### 2.3.3 Logistic regression branch

The raw-waveform Logistic Regression branch provides a linear decision boundary over the complete GRF trial and does not use predefined biomechanical descriptors. Its input is the same 3×101 waveform used by the neural branches, flattened as

$$r=Flatten\left(x\right)\in R^{303}.$$ (16)

The pathological-class probability is computed as

$$p^{\text{LR}}=\sigma \;\left(w_{\text{LR}}^{⊤}r+b_{\text{LR}}\right),$$ (17)

where σ(⋅) denotes the sigmoid function.

#### 2.3.4 Hybrid screening score

The final GaitScreen score is obtained by equal-weight probability averaging, a simple non-trainable fusion rule:

$$s_{i}=\frac{1}{3}\left(p_{i}^{GS}+p_{i}^{\mathrm{CNN}}+p_{i}^{LR}\right).$$ (18)

The threshold-dependent review flag is defined as

$${\hat{r}}_{i}\left(\tau \right)=\left\{\begin{array}{cc} 1, & s_{i}\geq \tau ,\\ 0, & s_{i}<\tau , \end{array}\right.$$ (19)

where τ is selected on the validation set. Thus, $s_{i}$ represents the GaitScreen score on a probability scale, while ${\hat{r}}_{i}$ represents the corresponding review flag.

### 2.4 Comparator models

To evaluate GaitScreen against representative alternatives, comparator models were selected to cover classical machine learning, standard deep learning, modern temporal architectures, and literature-inspired GRF models. The benchmark included.

- *Classical Machine Learning*: Logistic Regression, Support Vector Machine with an RBF kernel (SVM-RBF), and Random Forest (Cortes and Vapnik, 1995; Breiman, 2001);
- *Standard Deep Learning*: Multi-Layer Perceptron (MLP), CNN, LSTM, and Transformer models (Hochreiter and Schmidhuber, 1997; Vaswani et al., 2017);
- *Advanced Temporal Architectures*: Temporal Convolutional Network (TCN), ResNet1D, and InceptionTime (Bai et al., 2018; Wang et al., 2017; Ismail Fawaz et al., 2020);
- *Literature-Inspired Comparators*: model families adapted from previously reported GRF-based approaches, including Slijepcevic-style PCA-RBF SVM, GaitRec-Net-style 1D CNN, GMM-LIME-study MLP, Jani-style boosted trees, and GRF-Former-style Transformer models (Slijepcevic et al., 2018; Pandey et al., 2022; Jani et al., 2022; Mulwa et al., 2024; Wang et al., 2025). These models were evaluated under the present protocol and are not exact reproductions of the published systems.

All comparator models were evaluated using the identical subject-exclusive train/validation/test partition, GRF-only input representation, and trial-level healthy-control-versus-pathological-gait reference status. This design supported a unified internal comparison of model families; it was not a direct comparison with published performance values obtained under different tasks, inputs, and validation protocols.

Neural comparator models were trained from scratch using class-weighted cross-entropy and Adam optimisation, with learning rate of $10^{-3}$ , weight decay of $10^{-4}$ , batch size of 64, and early stopping with patience of 8 over a maximum of 50 epochs. Classical estimators and tree-based models were trained on flattened raw GRF waveforms, with model-specific balancing or standardisation where applicable. Each stochastic single-model comparator was evaluated over ten matched seeds, 42–51, with the decision threshold selected separately on the validation set for each seed by maximising macro-F1 before being applied once to the held-out test set. GaitScreen was evaluated over the same ten matched seeds by averaging the pathological-class probabilities from the multi-scale temporal backbone, CNN, and Logistic Regression components for each seed, selecting the decision threshold on the validation set, and applying the selected threshold once to the held-out test set. Detailed comparator architectures, training settings, and hyperparameters are provided in Supplementary Table S1.

### 2.5 Ablation design

Ablation analyses were conducted to examine whether the main design choices in GaitScreen contributed to the final screening scores. Two aspects were evaluated: the temporal-receptive-field configuration of the multi-scale temporal backbone and the contribution of each probability component in the hybrid fusion.

First, a backbone ablation was used to assess the effect of temporal scale selection. The selected multi-scale temporal backbone uses parallel one-dimensional convolutional stems with kernel sizes (3,13,25), followed by two lightweight convolutional encoding stages. To test this design choice, we compared the selected configuration with alternative backbone variants, including a raw-waveform variant without the multi-scale stem, a single-scale (25) variant, an alternative multi-scale (13,25,51) variant, and an all-kernel variant using all candidate temporal kernels considered in the design search. These variants were evaluated under the same subject-exclusive partition and validation-threshold procedure as the main model.

Second, a hybrid-fusion ablation was used to assess the contribution of the three probability components used by GaitScreen. The multi-scale temporal backbone, CNN, and Logistic Regression components were evaluated as individual predictors and as equal-average probability combinations. The primary ablation compared the backbone-only model, the backbone-plus-CNN fusion, and the full GaitScreen fusion comprising the multi-scale temporal backbone, CNN, and Logistic Regression components. Expanded hybrid-fusion settings were evaluated as supplementary analyses.

### 2.6 Model evaluation

#### 2.6.1 Screening performance metrics

Model evaluation was designed to characterise both overall discrimination and threshold-dependent screening behaviour. The evaluation metrics are defined in Equations 20–22. Threshold-independent discrimination was quantified using the area under the receiver operating characteristic curve (AUROC). For a selected decision threshold, threshold-dependent performance was evaluated using accuracy, macro-F1, sensitivity, specificity, false-positive rate (FPR), and false-negative rate (FNR). Given true positives (TP), true negatives (TN), false positives (FP), and false negatives (FN), accuracy was defined as

$$Accuracy=\frac{TP+TN}{TP+TN+FP+FN}.$$ (20)

Sensitivity and specificity were defined as

$$\begin{array}{cc} Sensitivity & =\frac{TP}{TP+FN},\\ Speci\mathrm{fi}city & =\frac{TN}{TN+FP}. \end{array}$$ (21)

The false-positive and false-negative rates were defined as

$$\begin{array}{cc} FPR & =\frac{FP}{FP+TN},\\ FNR & =\frac{FN}{FN+TP}. \end{array}$$ (22)

Macro-F1 was computed as the unweighted mean of the class-wise F1-scores, providing a class-balanced summary of thresholded performance. FNR reflects pathological-gait trials not flagged for review, whereas FPR reflects healthy-control trials flagged for review.

#### 2.6.2 Computational efficiency metrics

Computational complexity was characterised using fitted parameter count, serialised model size, single-sample CPU inference latency, and inference throughput. Parameter counts included all trainable parameters in the neural models and the fitted coefficients and intercept of the raw-waveform Logistic Regression component; classical estimators without a comparable cross-family parameter definition are marked with a dash in Table 1. The serialised size of GaitScreen was measured for the complete fitted model, including both neural components and the Logistic Regression component. The timing benchmark was conducted on an Apple M4 CPU using time-normalised 3×101 GRF inputs. Single-sample latency was measured at batch size 1 over 1000 repetitions after 50 warm-up passes, with gradient computation disabled. Each latency measurement included prediction by the multi-scale temporal backbone, CNN, and raw-waveform Logistic Regression components, conversion to pathological-class probabilities, equal probability averaging, and thresholding. Input preparation and model initialisation were completed before timing. Throughput was measured independently using batches of 64 over 100 repetitions after five warm-up batches. Accordingly, latency and throughput quantify model inference after GRF preprocessing rather than the complete acquisition-to-decision workflow.

**TABLE 1:** Model complexity and post-preprocessing inference efficiency under the common CPU benchmark.

Model	Params	Size (MB)	Latency (ms)	Throughput (samples/s)
LogReg	–	0.010	0.142 ± 0.025	136,218
SVM-RBF	–	4.875	0.364 ± 0.044	4,672
RandomForest	–	27.953	26.993 ± 3.265	2,042
MLP	110,978	0.423	0.021 ± 0.009	1,225,085
CNN	142,466	0.547	0.194 ± 0.042	840
LSTM	200,450	0.765	1.821 ± 0.096	3,019
Transformer	67,330	0.282	0.341 ± 0.059	7,077
TCN	426,306	1.633	1.006 ± 0.113	592
ResNet1D	480,386	1.841	0.642 ± 0.072	222
InceptionTime	492,418	1.884	1.116 ± 0.257	347
FGD-SVM	–	0.395	0.264 ± 0.002	14,660
GaitRec-net	302,402	1.157	0.158 ± 0.002	1,156
GMM-LIME	–	0.219	0.146 ± 0.001	316,601
EGA-CatBoost	–	1.406	5.806 ± 1.000	11,397
GRF-former	207,042	0.809	0.721 ± 0.013	3,232
GaitScreen	285,832	1.122	0.626 ± 0.010	484

Parameter counts are reported when a comparable fitted-parameter definition is available; dashes denote classical estimators for which this count was not used. Size denotes the serialised model size, not runtime memory. Latency is mean ± SD for time-normalised inputs at batch size 1; throughput was measured separately at batch size 64. All models were measured under the same benchmark; values for literature-inspired comparators should not be interpreted as runtime claims for the originally published systems.

#### 2.6.3 Statistical analysis

For the main trial-level comparison, the fixed partition was adopted because the cohort size permitted separate subject-exclusive training, validation, and held-out test subsets while retaining 1,228 trials for independent testing. Applying the same partition to every model also provided a common comparison set without variation from alternative subject allocations. All models were evaluated over ten matched seeds under this partition. Performance was reported as mean ± standard deviation across runs; this variability reflects model refitting and training stochasticity under the fixed partition. The final GaitScreen was used as the statistical reference and was compared with the baseline models using paired t -tests over matched run-level differences for Accuracy, macro-F1, and AUROC.

### 2.7 Interpretability analysis

Model interpretability analyses were conducted on the multi-scale temporal backbone component of GaitScreen and comprised SHAP-based attribution and t-SNE representation analysis.

#### 2.7.1 SHAP

SHapley Additive exPlanations (SHAP) (Lundberg and Lee, 2017) were applied after model fitting to the trained multi-scale temporal backbone. The analysis quantified the contribution of individual time samples and channels to the pathological-class logit within the predefined vertical, anterior–posterior, and mediolateral GRF input; it was not used to select the input channels. GradientExplainer used a stratified subset of 100 training trials as the background reference set, and explanations were generated for held-out test trials in mini-batches. The resulting attribution maps were aggregated as mean absolute SHAP values across trials to summarise channel- and stance-phase-specific contributions to the backbone output.

#### 2.7.2 t-SNE

Penultimate-layer features were extracted from the fitted representative multi-scale temporal backbone as the flattened encoder output for each held-out test trial. These features were projected into two dimensions using t-distributed stochastic neighbour embedding (t-SNE) (van der Maaten and Hinton, 2008). The t-SNE analysis used perplexity 30, PCA initialisation, automatic learning-rate selection, 1000 optimisation iterations, and a random state matched to the representative model seed. No additional feature standardisation or PCA dimensionality reduction was applied before t-SNE; PCA was used only to initialise the embedding. Because t-SNE is a nonlinear two-dimensional projection, the resulting geometry was interpreted qualitatively and does not preserve all relationships in the original high-dimensional representation.

## 3 Results

### 3.1 Trial-level screening performance comparison

Table 2 summarises the trial-level performance of GaitScreen and all comparator models under the unified subject-exclusive GaitRec protocol. Threshold-independent discrimination was assessed using AUROC, while threshold-dependent performance was assessed at the validation-selected review threshold using accuracy and macro-F1.

**TABLE 2:** Trial-level screening performance comparison under the subject-exclusive GaitRec protocol.

Model	Accuracy	Macro-F1	AUROC
LogReg	0.8581 ± 0.0027***	0.8579 ± 0.0027***	0.9362 ± 0.0002***
SVM-RBF	0.8546 ± 0.0023***	0.8532 ± 0.0023***	0.9214 ± 0.0003***
RandomForest	0.8461 ± 0.0038***	0.8432 ± 0.0043***	0.9233 ± 0.0005***
MLP	0.8463 ± 0.0014***	0.8443 ± 0.0016***	0.9202 ± 0.0017***
CNN	0.8600 ± 0.0088**	0.8583 ± 0.0095**	0.9366 ± 0.0036***
LSTM	0.7453 ± 0.0843**	0.7404 ± 0.0868**	0.8270 ± 0.1199*
Transformer	0.8511 ± 0.0081***	0.8502 ± 0.0082***	0.9296 ± 0.0023***
TCN	0.8652 ± 0.0080**	0.8633 ± 0.0086**	0.9378 ± 0.0023***
ResNet1D	0.8621 ± 0.0047***	0.8607 ± 0.0048***	0.9351 ± 0.0033***
InceptionTime	0.8621 ± 0.0128**	0.8597 ± 0.0132**	0.9374 ± 0.0102
FGD-SVM	0.8379 ± 0.0024***	0.8366 ± 0.0023***	0.9135 ± 0.0005***
GaitRec-net	0.8555 ± 0.0069***	0.8550 ± 0.0067***	0.9333 ± 0.0038***
GMM-LIME	0.8169 ± 0.0196***	0.8163 ± 0.0192***	0.9004 ± 0.0135***
EGA-CatBoost	0.8306 ± 0.0085***	0.8301 ± 0.0083***	0.9145 ± 0.0020***
GRF-former	0.8454 ± 0.0037***	0.8442 ± 0.0039***	0.9240 ± 0.0026***
**GaitScreen**	**0.8766** ± **0.0025**	**0.8757** ± **0.0024**	**0.9425** ± **0.0015**

Values are mean ± standard deviation over ten matched runs. Superscripts indicate comparator models significantly lower than GaitScreen after paired t -tests (* p<0.05 , ** p<0.01 , *** p<0.001 ).

Bold values indicate the best performance for each evaluation metric.

GaitScreen achieved the highest mean performance across all three primary metrics, with an AUROC of 0.9425±0.0015 , a thresholded test-set accuracy of 0.8766±0.0025 , and a macro-F1 of 0.8757±0.0024 . Among the classical machine-learning models, Logistic Regression was the strongest comparator. Among the standard and advanced neural-network baselines, TCN and InceptionTime were the closest competitors, but both remained below GaitScreen in mean accuracy, macro-F1, and AUROC.

All literature-inspired comparators obtained lower mean performance than GaitScreen under the present protocol. The GaitRec-Net-style model was the strongest of these comparators, but its AUROC and macro-F1 remained below those of GaitScreen.

Paired statistical comparisons used GaitScreen as the reference model. GaitScreen was significantly higher than all comparator models for accuracy and macro-F1. For AUROC, GaitScreen was significantly higher than 14 of the 15 comparator models; InceptionTime had a lower mean AUROC than GaitScreen, but the AUROC difference was not statistically significant.

### 3.2 Threshold-dependent screening behaviour

Figure 2 shows the test-set receiver operating characteristic curve of the final GaitScreen score, averaged over ten matched runs. The curve indicates strong threshold-independent discrimination, with a mean AUROC of 0.9425±0.0015 . The marker indicates the test-set operating point obtained after applying the validation-selected max macro-F1 review threshold, with a mean threshold of τ=0.528 .

**FIGURE 2:**
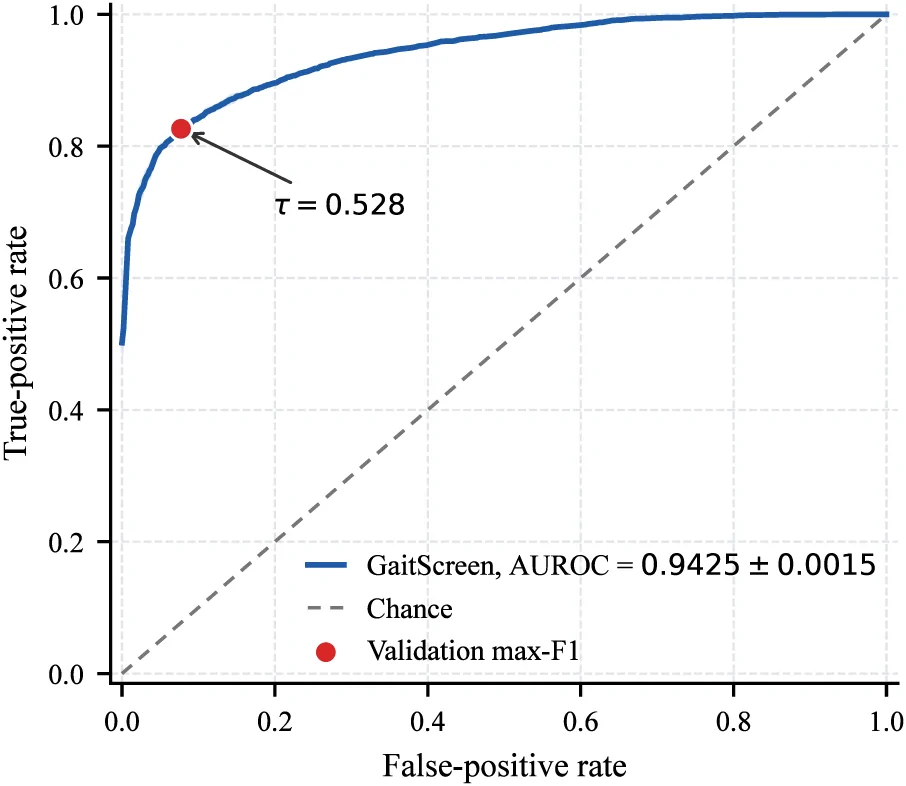
Test-set receiver operating characteristic (ROC) curve of the final GaitScreen score. The curve shows the mean ROC over ten matched runs, and the shaded band indicates one standard deviation. The marker indicates the test-set operating point obtained using the validation-selected max macro-F1 review threshold. The mean AUROC was 0.9425±0.0015 .

At this validation-selected review threshold, GaitScreen produced a balanced profile, with a false-negative rate of 0.1736. Using the high-sensitivity threshold, the false-negative rate decreased to 0.1073, with more false-positive review flags. The fixed 0.5, validation-selected max macro-F1, high-sensitivity, and Youden operating points are reported in Supplementary Table S2.

Pathology-family analysis showed that screening behaviour varied across anatomical groups. Performance was strongest for calcaneus trials, whereas knee trials had the lowest sensitivity and the highest false-negative rate among the pathology families. This indicates that the pooled pathological screening task did not have uniform difficulty across anatomical pathology families. Full family-wise results are reported in Supplementary Table S3.

### 3.3 Model compactness and inference efficiency

Table 1 summarises model complexity and post-preprocessing inference efficiency under the common CPU benchmark. The full hybrid GaitScreen model contained 285,832 fitted parameters and had a serialised size of 1.122 MB. It was larger than the raw-waveform Logistic Regression and several compact neural comparators, but smaller by parameter count and serialised size than the evaluated TCN, ResNet1D, and InceptionTime models. On the Apple M4 CPU, full hybrid inference required 0.626±0.010 ms per time-normalised GRF trial at batch size 1 and processed 483.7 samples/s in the separate batch-size-64 throughput measurement. The latency estimate covers the multi-scale temporal backbone, CNN, raw-waveform Logistic Regression, probability averaging, and thresholding after GRF preprocessing.

Model compactness is relevant to post-acquisition trial review because the score is generated after GRF preprocessing. Previous GRF-based studies have demonstrated strong performance using CNN-based, ensemble, explainable-learning, and Transformer-style models (Pandey et al., 2022; Jani et al., 2022; Mulwa et al., 2024; Wang et al., 2025).

Under the common evaluation and timing protocol, GaitScreen occupied an intermediate complexity regime. It required more parameters, storage, and inference work than the raw-waveform Logistic Regression and some compact neural models, while remaining smaller by parameter count and serialised size than the evaluated TCN, ResNet1D, and InceptionTime models. GaitScreen also achieved the strongest mean trial-level performance in the fixed-partition, ten-run comparison (accuracy 0.8766±0.0025 , macro-F1 0.8757±0.0024 , and AUROC 0.9425±0.0015 ). The result is therefore a performance–complexity trade-off rather than a claim that GaitScreen is the minimum-complexity model.

All numerical complexity values in Table 1 refer to models evaluated under this common benchmark. Literature-inspired comparators were adapted and evaluated under the present protocol; their measurements should not be attributed directly to the originally published systems. Direct numerical runtime comparison across publications is not appropriate when hardware, input representations, preprocessing, model specifications, and timing scopes differ.

### 3.4 Post-hoc subject-level aggregation and calibration

Table 3 summarises the *post hoc* subject-level aggregation and calibration results. Many GRF-based gait-classification studies report trial-level or sample-level performance (Slijepcevic et al., 2018; Pandey et al., 2022; Jani et al., 2022; Mulwa et al., 2024; Wang et al., 2025); the present sensitivity analysis examines how the trial scores behave after within-subject aggregation.

**TABLE 3:** Post-HOC subject-level aggregation and calibration analysis.

Analysis	Macro-F1	AUROC	Sens	Spec	ECE
Trial-level screening scores	0.8757	0.9425	0.8264	0.9228	0.0375
Subject mean-screening-score aggregation	0.7652	0.9530	0.9500	0.6000	0.1733
Subject majority-vote aggregation	0.7645	0.9103	0.8810	0.9065	–

Values are mean values over ten matched runs. ECE, denotes expected calibration error.

Post-hoc mean-score aggregation preserved strong subject-level ranking, but it changed the sensitivity–specificity profile and increased calibration error. The aggregated score should therefore not be interpreted as a calibrated subject-level risk estimate.

The primary validated claim remains trial-level classification and review prioritisation. Subject-level aggregation is reported only as a *post hoc* sensitivity analysis, consistent with guidance that model outputs should be validated at their intended decision level (Collins et al., 2024; Vasey et al., 2022; Sperrin et al., 2022).

### 3.5 Ablation and hybrid-fusion analysis

Table 4 reports the temporal-scale ablation and hybrid-fusion analysis of GaitScreen, using trial-level macro-F1 as the performance readout. In the temporal-scale ablation, the selected {3,13,25} multi-scale backbone achieved the highest macro-F1 among the tested backbone variants. It improved over the raw-waveform variant and performed slightly better than the single-scale, alternative multi-scale, and all-kernel configurations. This supports the use of local and medium-range receptive fields for encoding stance-phase GRF patterns.

**TABLE 4:** Temporal-scale ablation and hybrid-fusion analysis of gaitscreen.

Multi-scale backbone					
Variant	Raw	{25}	{13,25,51}	{3,13,25}	All
Macro-F1	0.8583	0.8666	0.8669	**0.8670**	0.8649

Hybrid fusion			
Backbone	CNN	LR	Macro-F1
✓	×	×	0.8670
✓	✓	×	0.8687
✓	✓	✓	**0.8757**

Bold values indicate the highest Macro‐F1 score within each ablation analysis.

The hybrid-fusion ablation further showed that combining complementary components improved the final screening scores. The backbone-only model achieved a macro-F1 of 0.8670, the backbone-plus-CNN fusion increased macro-F1 to 0.8687, and the full GaitScreen fusion with the multi-scale temporal backbone, CNN, and Logistic Regression components achieved the highest macro-F1 of 0.8757. These results indicate that the final screening scores benefited from combining learned temporal features, conventional convolutional features, and raw-waveform linear information.

Expanded hybrid-fusion results, including all single-component, pairwise-fusion, full-fusion, and calibrated-average variants, are reported in Supplementary Table S4.

### 3.6 Feature attribution and interpretability analysis

Figure 3 shows where the fitted multi-scale temporal backbone assigned attribution within its predefined three-component GRF input. Mean absolute SHAP values were concentrated in particular channels and stance-phase regions rather than distributed uniformly across the waveform. Vertical GRF showed the largest mean absolute attribution, with its strongest region in late stance; anterior–posterior and mediolateral GRF also showed localised attribution over the stance cycle. These findings describe channel- and phase-specific reliance of the fitted backbone.

**FIGURE 3:**
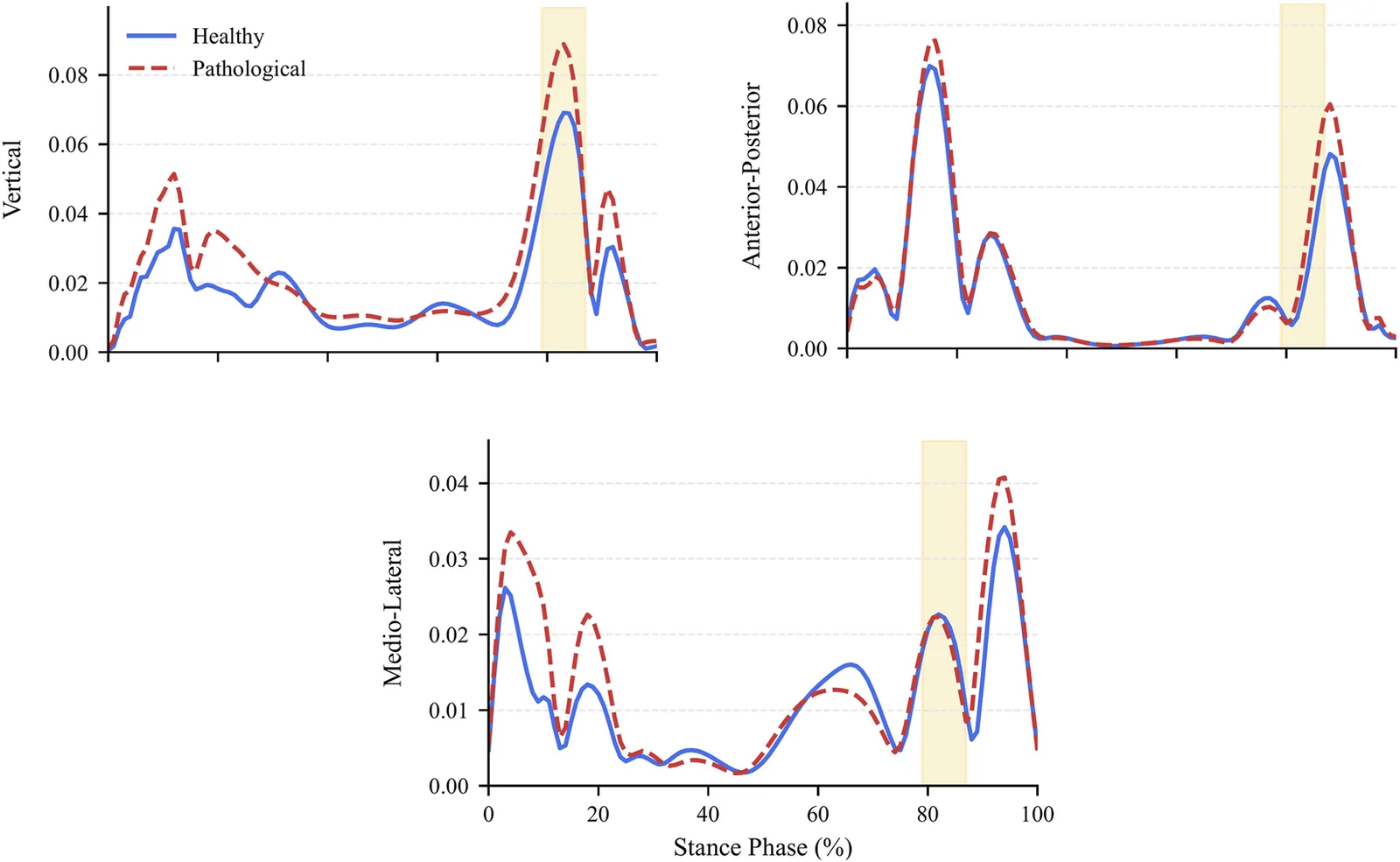
Feature attribution of the multi-scale temporal backbone component across the three GRF channels. Mean absolute SHAP values were computed for the pathological-class logit and aggregated over held-out test trials. The attribution map highlights channel- and stance-phase-specific GRF measurements that contributed most strongly to the pathological-gait screening output of the backbone component.

Figure 4 shows the t-SNE projection of held-out penultimate-layer features from the fitted representative multi-scale temporal backbone. Healthy-control and pathological-gait trials occupied partially distinct but overlapping regions. The projection therefore suggests class-associated organisation in the learned backbone representation, while the substantial residual overlap indicates that the two classes were not completely separable in two dimensions. This overlap is consistent with the trial-level binary task and the heterogeneity of the pooled lower-limb orthopaedic pathological class. Quantitative performance conclusions are based on held-out predictions and the reported evaluation metrics rather than on the visual appearance of the t-SNE projection.

**FIGURE 4:**
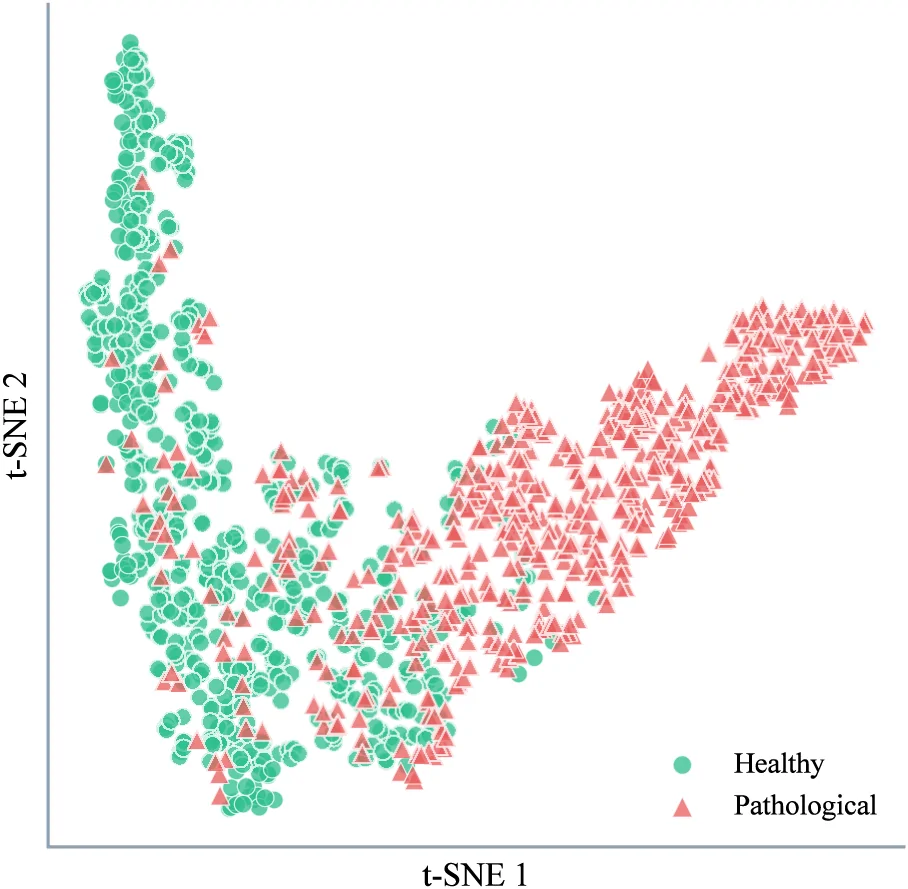
t-SNE visualisation of penultimate-layer features extracted from the multi-scale temporal backbone component on the held-out test set. The projection provides a qualitative view of class-related organisation in the learned GRF representation space, while residual overlap reflects the heterogeneous trial-level healthy-control-versus-pathological-gait screening task.

## 4 Discussion

This study evaluated GaitScreen as a compact, post-acquisition framework for prioritising recorded GRF trials. The model combines a multi-scale temporal backbone, a CNN component, and a raw-waveform Logistic Regression component through equal-weight probability averaging. Under the subject-exclusive GaitRec protocol, GaitScreen achieved the strongest mean trial-level performance among the evaluated models. The analysis also characterised review-threshold behaviour, pathology-family variation, computational efficiency, component and fusion effects, and *post hoc* aggregation and calibration.

### 4.1 Trial-level screening performance

Previous GRF-based studies have shown that stance-phase force trajectories contain strong discriminative information for pathological gait, using approaches such as PCA-SVM pipelines, CNN-based GaitRec models, ensemble classifiers, explainable-learning frameworks, and Transformer-style architectures (Slijepcevic et al., 2018; Pandey et al., 2022; Jani et al., 2022; Mulwa et al., 2024; Wang et al., 2025). The present findings are consistent with this evidence, as GRF signals alone supported strong healthy-control-versus-pathological-gait screening performance.

Within the common internal evaluation, GaitScreen outperformed the evaluated classical, deep-learning, and literature-inspired reference models. This suggests that multi-scale temporal, conventional convolutional, and raw-waveform linear information are complementary for the present trial-level task. The adapted literature-inspired comparators are not exact reproductions of published systems.

### 4.2 Threshold-dependent referral behaviour

The threshold-dependent analysis complements AUROC by showing how the selected operating point affects the review flags. The validation-selected max macro-F1 threshold provided a balanced profile, whereas the high-sensitivity threshold reduced the false-negative rate at the cost of more false-positive flags.

The pathology-family analysis showed that a single pooled threshold did not behave uniformly across anatomical groups. Knee trials had lower sensitivity, indicating that subgroup performance should be examined when interpreting the pooled result.

### 4.3 Model compactness and practical screening feasibility

GaitScreen achieved the strongest mean trial-level performance under the fixed-partition protocol while remaining in an intermediate complexity regime, with 285,832 fitted parameters, a 1.122 MB serialised size, and a single-sample CPU inference latency of 0.626±0.010 ms after GRF preprocessing.

For fixed-length standardised stance-phase GRF trajectories, the results show a performance–complexity trade-off relative to the evaluated linear and temporal models (Pandey et al., 2022; Jani et al., 2022; Mulwa et al., 2024; Wang et al., 2025).

The compact model size and low inference latency support computational scalability for post-acquisition processing of standardised GRF trials in settings where force-plate acquisition is already available. The scalability of data acquisition and end-to-end clinical deployment remains to be evaluated.

### 4.4 Subject-level aggregation and calibration

The ablation results support the two main design choices in GaitScreen. Among the evaluated temporal configurations, the 3, 13, 25 multi-scale backbone achieved the highest macro-F1, suggesting that combining local and medium-range receptive fields was more effective than the raw-waveform, single-scale, alternative multi-scale, and all-kernel variants under the adopted protocol.

The module-level analysis further showed incremental improvements from the backbone-only model to the backbone-plus-CNN fusion and the full GaitScreen configuration. The complete fusion of the multi-scale backbone, CNN, and Logistic Regression components achieved the highest macro-F1. These results support the use of complementary temporal, convolutional, and linear waveform representations, although the observed gains should be interpreted within the current internal dataset and evaluation protocol.

### 4.5 Feature attribution and model interpretability

The three GRF components were included before model training because they describe complementary directions of stance-phase force: vertical GRF primarily reflects body support and loading, anterior–posterior GRF captures braking and propulsion, and mediolateral GRF reflects side-to-side force regulation and aspects of frontal-plane control (Whittle, 1996; Baker, 2006; Simon, 2004; Winter 1991). The subsequent SHAP analysis addressed a different question: where the fitted backbone used information within that predefined input space.

Within the fitted backbone, vertical GRF showed the largest mean absolute attribution, with the strongest vertical region in late stance. The localised anterior–posterior and mediolateral attributions indicate that the backbone also used directional information across the stance cycle. Because SHAP was applied only to this component, the attribution does not explain the final fused GaitScreen probability.

The t-SNE projection in Figure 4 showed partial class-associated organisation with substantial overlap in the backbone representation. It is a qualitative representation analysis; quantitative conclusions are based on held-out prediction metrics.

### 4.6 Limitations and future work

Several limitations define the current scope of GaitScreen. First, although the evaluation used a subject-exclusive protocol, all experiments were conducted within the GaitRec dataset. Therefore, the results reduce subject-level information leakage within this benchmark, but do not establish external generalisation across laboratories, force-plate systems, acquisition protocols, or patient populations. External validation on independently collected GRF datasets is needed before the reported performance can be interpreted as generalisable beyond the present cohort.

The fixed subject-exclusive partition provided an independent held-out benchmark shared by all models, and the ten matched runs quantified refitting variability under this design. Repeated subject-exclusive repartitioning would provide complementary evidence on sensitivity to alternative subject allocations and may be considered in future validation.

All retained trials used GaitRec’s categorical self-selected-speed condition, but continuous trial-level walking velocity was unavailable. Pathology-related and speed-related waveform effects therefore cannot be fully disentangled.

Because of practical constraints, model efficiency was evaluated only in an offline setting.

Second, the review thresholds were selected retrospectively from the validation set. The high-sensitivity operating mode is a threshold-dependent sensitivity analysis rather than a prospectively fixed clinical rule.

Third, the primary validated claim remains trial-level. Post-hoc subject-level aggregation changed the operating characteristics and calibration, so the aggregated scores should not be interpreted as calibrated subject-level risk estimates.

Fourth, the binary healthy-control-versus-pathological-gait formulation does not distinguish pathology subtype, anatomical source, disease severity, or clinical aetiology. The family-wise results also show that performance differs across anatomical pathology groups.

Finally, this study used standardised laboratory GRF trajectories. The findings should not be assumed to transfer directly to other sensing systems or patient populations without external validation.

## 5 Conclusion

This study developed GaitScreen, a compact GRF framework that assigns post-acquisition review-prioritisation scores to recorded stance-phase GRF trials. Under the fixed subject-exclusive GaitRec protocol, it achieved the strongest mean trial-level performance among the evaluated models while maintaining a small serialised size and low CPU inference latency. The study also characterised review-threshold behaviour, pathology-family variation, component and fusion effects, and *post hoc* aggregation and calibration. The evidence supports computationally scalable post-acquisition analysis of standardised GRF trials.

## Data Availability

The original contributions presented in the study are included in the article/Supplementary Material, further inquiries can be directed to the corresponding author.
