# Supplementary material for: GaitScreen: a ground reaction force-based framework for prescreening gait abnormalities in orthopaedic populations
Source: Front Bioeng Biotechnol. 2026 Oct 26;14:1947887. doi: 10.3389/fbioe.2026.1947887 (PMC13745668; doi:10.3389/fbioe.2026.1947887)
Supplement: Supplementary file 1 [file Supplementaryfile1.pdf]

Supplementary Material

SUPPLEMENTARY METHOD S1. DATASET FILTERING AND PREPROCESSING

Experiments used the public GaitRec benchmark, which contains bilateral GRF recordings from 2,084 pathological participants and 211 healthy-control participants. The pathological cohort is organised into hip, knee, ankle, and calcaneus families with detailed subclass labels. For the binary task, retained labels were mapped to healthy-control and pathological-gait classes after filtering.

The retained data were restricted to sessions recorded at self-selected walking speed and in normal shoes. Both limbs were retained for healthy-control participants, whereas the clinically affected limb or limbs were retained for pathological participants according to the limb-level clinical metadata. Detailed pathology labels that could not support the adopted subject-exclusive train/validation/test allocation were excluded, leaving 23 detailed pathological subclasses. To avoid overweighting subjects with long rehabilitation histories, only the first eligible session for each subject was retained, with up to five stance-phase trials from each retained limb.

The final limb-level screening cohort comprised 2,189 subjects: 208 healthy-control subjects and 1,981 pathological subjects. The pathological cohort included hip ($n = 434$), knee ($n = 600$), ankle ($n = 590$), and calcaneus ($n = 357$) disorders. The resulting trial set contained 12,089 limb-specific trials, including 2,078 healthy-control trials and 10,011 pathological-gait trials.

No additional temporal resampling or handcrafted feature extraction was applied. Each retained trial was represented by the complete 3×101 GaitRec waveform: vertical, anterior–posterior, and mediolateral GRF, with 101 time-normalised stance-phase samples per channel and 303 values in total. GRF amplitudes are expressed as multiples of body weight, and directional signs are standardised such that anterior and medial forces are positive (Horsak et al., 2020).

For evaluation, the official GaitRec train–test split was not used. Subjects were allocated once to a subject-exclusive train/validation/test partition after fine stratification over retained detailed pathology subclasses and only then mapped to the healthy-control-versus-pathological-gait screening task. The held-out test set comprised 1,228 trials (640 healthy-control trials and 588 pathological-gait trials); the remaining subjects were used for training and validation under the same stratification rule. Healthy-control trials were oversampled with replacement during model development after the split.

SUPPLEMENTARY METHOD S2. MULTI-SCALE TEMPORAL BACKBONE AND HYBRID INFERENCE

The multi-scale temporal backbone uses three parallel one-dimensional convolutional branches with kernel sizes $\{3, 13, 25\}$ to encode complementary local and medium-range stance-phase GRF patterns. The branch outputs are concatenated and passed through two convolutional encoding stages: a 128-channel convolution with kernel size 5, followed by a 256-channel convolution with kernel size 3. Each encoding stage uses batch normalisation, GELU activation, and dropout. The encoded feature map is vectorised and mapped to healthy-control and pathological-gait logits by a linear output head.

All three GaitScreen components use the same complete 3×101 waveform. The multi-scale temporal backbone and CNN retain the channel-by-time representation, whereas the raw-waveform Logistic Regression component flattens it to 303 dimensions.

The selected backbone setting uses dropout probabilities 0.10, 0.15, and 0.25 across the compact backbone components, as summarised in Supplementary Table S1. The final GaitScreen screening scores are equal-weight averages of the pathological-class probabilities from matched multi-scale temporal backbone, CNN, and raw-waveform Logistic Regression components. The averaging rule does not introduce an additional trainable stacking model, and the final threshold is selected on validation data only before held-out test evaluation.

SUPPLEMENTARY TABLE S1. TRIAL-LEVEL MODEL ARCHITECTURES AND HYPERPARAMETERS

Table S1. Trial-level model architectures and hyperparameters, including representative models adapted from prior studies.

Model	Layers	Width / Hidden	Kernels / Heads	FF / Trees	Dropout	Learning Rate / Main Setting	Batch / Epochs
Raw-waveform Logistic Regression	–	303-d waveform input	–	–	0	balanced, 2000 iter	–
SVM-RBF	–	303-d input	–	RBF	0	C=1.0	–
Random Forest	500 trees	303-d input	–	500	0	balanced RF	–
MLP	2 FC + 1 FC	256, 128	–	–	0.20	10^{-3}	64 / 50
CNN	3 conv + 1 FC	64, 128, 256	7; 5; 3	–	0.30	10^{-3}	64 / 50
LSTM	2 LSTM + 1 FC	128	–	–	0.20	10^{-3}	64 / 50
Transformer	2 enc + 1 FC	d=64	4 heads	128	0	10^{-3}	64 / 50
TCN	3 tcn blks + 1 FC	64, 128, 256	dil 1/2/4	–	0.20	10^{-3}	64 / 50
ResNet1D	3 res blks + 1 FC	64, 128, 128	7; 5; 3	–	0	10^{-3}	64 / 50
InceptionTime	6 inc + 1 FC	32 / branch	11/21/41; bneck 32	–	0	10^{-3}	64 / 50
Slijepcevic-style PCA-RBF SVM	PCA + SVM	303-d input	PCA 20	RBF	0	C=1.0, γ=scale	–
GaitRec-Net-style 1D CNN	3 conv + 1 FC	100, 200, 400	3; 3; 3	–	0.50	10^{-3}	64 / 50
GMM-LIME-study MLP	2 FC + 1 FC	50, 50	–	–	0	tanh, α=0.01	–
Jani-style boosted trees	HGB trees	303-d input	–	300	0	lr 0.05, leaves 31	–
GRF-Former-style Transformer	4 enc + 1 FC	d=64	4 heads	256	0.10	10^{-3}	64 / 50
Multi-scale temporal backbone	3 br. + 2 conv + 1 FC	63, 128, 256	$\{3, 13, 25\}; 5; 3$	–	0.10/0.15/0.25	10^{-3}	64 / 50

SUPPLEMENTARY TABLE S2. FULL SCREENING OPERATING CHARACTERISTICS OF GAITSCREEN

Table S2. Full screening operating characteristics of GaitScreen. Values are mean values over ten matched runs. Thresholds other than fixed 0.5 were selected on validation data only.

Operating point	τ	Sensitivity	Specificity	PPV	NPV	FPR	FNR	macro-F1	Accuracy
Fixed 0.5	0.5000	0.8371	0.9084	0.8940	0.8587	0.0916	0.1629	0.8736	0.8743
Max macro-F1	0.5280	0.8264	0.9228	0.9082	0.8528	0.0772	0.1736	0.8757	0.8766
High sensitivity	0.3438	0.8927	0.8066	0.8093	0.8911	0.1934	0.1073	0.8478	0.8478
Youden	0.4783	0.8434	0.8962	0.8847	0.8627	0.1038	0.1566	0.8703	0.8709

SUPPLEMENTARY METHOD S3. SUBJECT-LEVEL AGGREGATION AND CALIBRATION

Because several correlated trials can come from the same participant, we performed a post-hoc subject-level sensitivity analysis outside the main trial-level claims. For each matched run, GaitScreen trial-level scores were averaged within each held-out subject, and a subject-level threshold was selected on validation subjects only by maximising macro-F1 before being applied once to the held-out test subjects. A majority-vote variant based on trial-level review flags was also evaluated.

The compact subject-level aggregation and calibration metrics are reported in the main text, while the calibration curves are retained in Supplementary Fig. S1. Subject-level mean screening scores were useful for ranking but should not be treated as externally calibrated absolute risks without additional validation.

SUPPLEMENTARY FIGURE S1. CALIBRATION CURVES

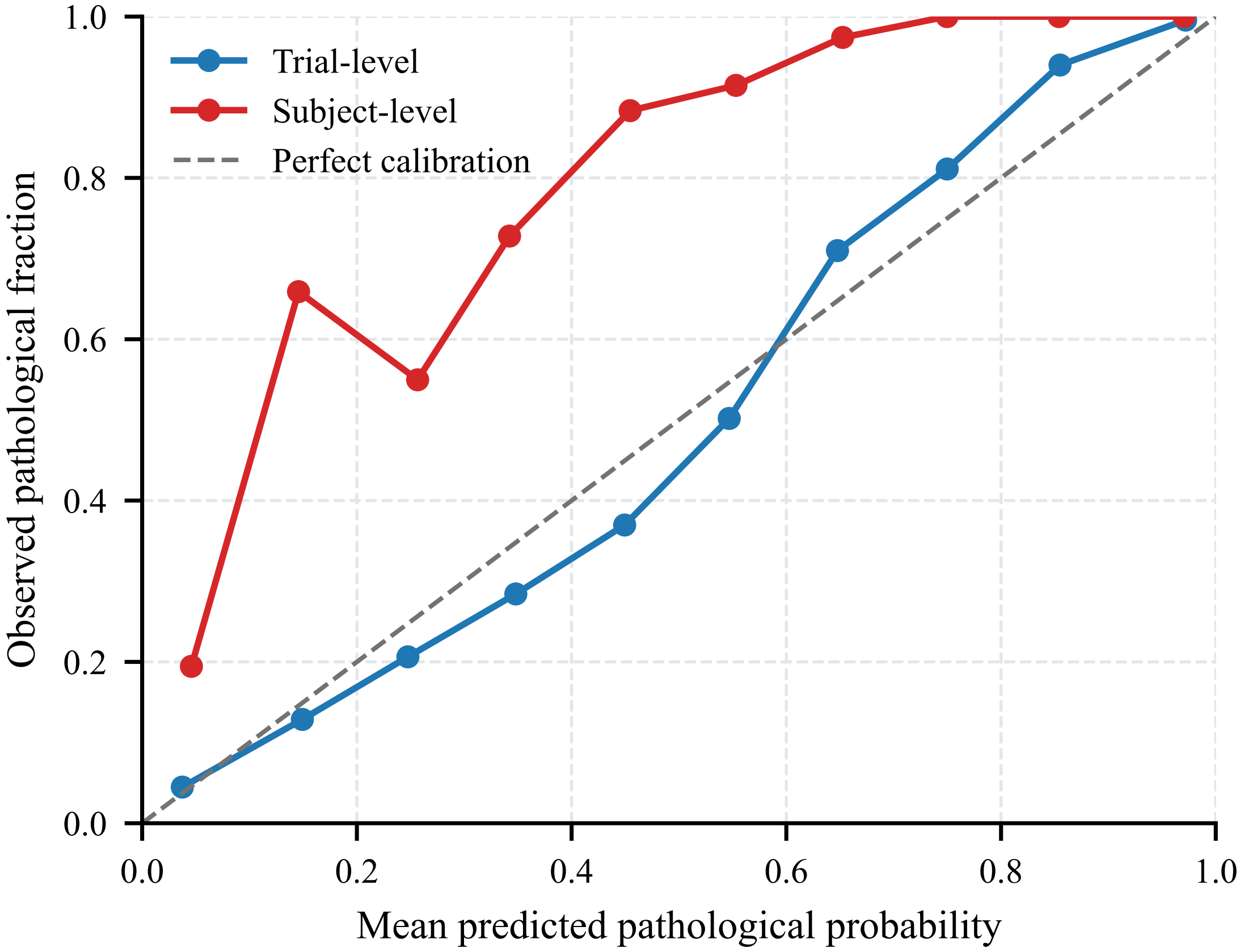

Figure S1. Calibration curves for final GaitScreen screening scores at trial and subject levels. Curves are ten-bin reliability diagrams averaged over ten matched runs. Trial-level calibration was moderate (Brier score 0.0929, ECE 0.0375), whereas subject-level aggregation increased ECE to 0.1733.

SUPPLEMENTARY TABLE S3. PATHOLOGY-FAMILY SCREENING BEHAVIOUR

Table S3. Family-wise GaitScreen screening behaviour in the held-out test set. AUROC is computed for each pathology family versus healthy-control trials. Sensitivity is computed within each pathology family using the validation-selected GaitScreen operating thresholds. Values are mean ± standard deviation over ten matched runs.

Family	Trials	Subjects	AUROC vs. healthy-control trials	Sensitivity at max-F1	FNR at max-F1	Sensitivity at high-sensitivity
Hip	130	65	0.9455 ± 0.0018	0.8377 ± 0.0156	0.1623 ± 0.0156	0.9039 ± 0.0054
Knee	176	88	0.9228 ± 0.0023	0.7625 ± 0.0212	0.2375 ± 0.0212	0.8534 ± 0.0079
Ankle	174	87	0.9342 ± 0.0018	0.8190 ± 0.0168	0.1810 ± 0.0168	0.8742 ± 0.0074
Calcaneus	108	54	0.9844 ± 0.0010	0.9287 ± 0.0107	0.0713 ± 0.0107	0.9732 ± 0.0068

SUPPLEMENTARY TABLE S4. HYBRID-FUSION ABLATION

Table S4. Hybrid-fusion ablation for the final GaitScreen screening scores. Values are mean ± standard deviation over ten matched runs; thresholds were selected on validation data only by maximising macro-F1.

Model	Fusion	Accuracy	macro-F1	AUROC	Sensitivity	Specificity
Multi-scale temporal backbone	Single	0.8685 ± 0.0047	0.8670 ± 0.0048	0.9382 ± 0.0042	0.8012 ± 0.0321	0.9303 ± 0.0303
CNN	Single	0.8600 ± 0.0088	0.8583 ± 0.0095	0.9366 ± 0.0036	0.7869 ± 0.0311	0.9272 ± 0.0167
Raw-waveform Logistic Regression	Single	0.8581 ± 0.0027	0.8579 ± 0.0027	0.9362 ± 0.0002	0.8597 ± 0.0076	0.8566 ± 0.0119
Multi-scale temporal backbone + CNN	Equal average	0.8699 ± 0.0061	0.8687 ± 0.0063	0.9399 ± 0.0023	0.8102 ± 0.0270	0.9248 ± 0.0213
Multi-scale temporal backbone + raw-waveform Logistic Regression	Equal average	0.8687 ± 0.0080	0.8683 ± 0.0077	0.9417 ± 0.0015	0.8568 ± 0.0234	0.8797 ± 0.0362
CNN + raw-waveform Logistic Regression	Equal average	0.8678 ± 0.0044	0.8675 ± 0.0042	0.9418 ± 0.0017	0.8534 ± 0.0169	0.8811 ± 0.0221
GaitScreen	Equal average	0.8766 ± 0.0025	0.8757 ± 0.0024	0.9425 ± 0.0015	0.8263 ± 0.0151	0.9228 ± 0.0159
GaitScreen, calibrated average	Validation isotonic	0.8686 ± 0.0066	0.8677 ± 0.0064	0.9420 ± 0.0016	0.8263 ± 0.0389	0.9075 ± 0.0437

SUPPLEMENTARY METHOD S4. FEATURE-BASED AND LEARNED-FUSION COMPARISONS

The biomechanical-feature Logistic Regression baseline (Bio24-LR) used 24 predefined descriptors extracted from the same three time-normalised GRF channels: eight vertical, eight anterior–posterior, and eight mediolateral descriptors. The features were standardised using training data, and the minority training class was oversampled with replacement for each matched seed. Logistic Regression used $C = 1$, balanced class weights, and a maximum of 2000 iterations. The decision threshold was selected on the validation set by maximising macro-F1 and then applied once to the held-out test set. Bio24-LR is separate from the raw-waveform Logistic Regression component of GaitScreen, which uses all 303 waveform values.

Two learned fusion rules were fitted to the validation-set probabilities from the matched multi-scale temporal backbone, CNN, and raw-waveform Logistic Regression components. Validation-learned convex weighting constrained three non-negative weights to sum to one and minimised validation log loss. Logistic stacking standardised the three component probabilities and fitted a balanced Logistic Regression meta-classifier with $C = 1$. A validation-selected macro-F1 threshold was determined separately for each fusion rule and seed. The test labels were not used to fit fusion parameters or select thresholds.

SUPPLEMENTARY TABLE S5. BIOMECHANICAL-FEATURE DEFINITIONS

Table S5: Exact definitions of the 24 descriptors used by Bio24-LR. Force amplitudes are expressed as multiples of body weight, and all areas are integrated over normalised stance fraction rather than physical time.

No. / channel	Descriptor	Extraction rule	Units
1 / V	First peak amplitude	Maximum within 0–40% stance	BW multiple
2 / V	First peak location	Location of the maximum within 0–40% stance	% stance
3 / V	Mid-stance minimum amplitude	Minimum within 20–70% stance	BW multiple
4 / V	Mid-stance minimum location	Location of the minimum within 20–70% stance	% stance
5 / V	Second peak amplitude	Maximum within 60–100% stance	BW multiple
6 / V	Second peak location	Location of the maximum within 60–100% stance	% stance
7 / V	Normalised stance-curve area	Trapezoidal area under the force curve over normalised stance fraction $[0, 1]$	BW multiple × normalised stance fraction
8 / V	Root-mean-square magnitude	Square root of the mean squared force across 101 stance samples	BW multiple
9 / AP	Braking minimum amplitude	Minimum within 0–60% stance	BW multiple
10 / AP	Braking minimum location	Location of the minimum within 0–60% stance	% stance
11 / AP	Propulsion maximum amplitude	Maximum within 40–100% stance	BW multiple
12 / AP	Propulsion maximum location	Location of the maximum within 40–100% stance	% stance
13 / AP	Braking-to-propulsion transition	First negative-to-nonnegative zero crossing between the identified braking minimum and propulsion maximum, using linear interpolation; nearest-to-zero sample in that interval if no crossing occurs	% stance
14 / AP	Signed normalised stance-curve area	Trapezoidal signed area under the force curve over normalised stance fraction $[0, 1]$	BW multiple × normalised stance fraction
15 / AP	Absolute normalised stance-curve area	Trapezoidal area under the absolute force curve over normalised stance fraction $[0, 1]$	BW multiple × normalised stance fraction
16 / AP	Root-mean-square magnitude	Square root of the mean squared force across 101 stance samples	BW multiple
17 / ML	Minimum amplitude	Minimum across 0–100% stance	BW multiple
18 / ML	Minimum location	Location of the minimum across 0–100% stance	% stance
19 / ML	Maximum amplitude	Maximum across 0–100% stance	BW multiple
20 / ML	Maximum location	Location of the maximum across 0–100% stance	% stance
21 / ML	Amplitude range	Maximum minus minimum across 0–100% stance	BW multiple
22 / ML	Root-mean-square magnitude	Square root of the mean squared force across 101 stance samples	BW multiple
23 / ML	Signed normalised stance-curve area	Trapezoidal signed area under the force curve over normalised stance fraction $[0, 1]$	BW multiple × normalised stance fraction
24 / ML	Absolute normalised stance-curve area	Trapezoidal area under the absolute force curve over normalised stance fraction $[0, 1]$	BW multiple × normalised stance fraction

SUPPLEMENTARY TABLE S6. FEATURE-BASED AND LEARNED-FUSION RESULTS

Table S6. Additional feature-based and fusion comparisons. Values are mean ± standard deviation over ten matched runs under the same fixed subject-exclusive partition.

Model / fusion rule	Accuracy	macro-F1	AUROC	Sensitivity	Specificity
Bio24-LR	0.8406 ± 0.0013	0.8384 ± 0.0012	0.9315 ± 0.0002	0.7578 ± 0.0086	0.9166 ± 0.0085
GaitScreen, equal average	0.8766 ± 0.0025	$\mathbf{0.8757 \pm 0.0024}$	$\mathbf{0.9425 \pm 0.0015}$	0.8264 ± 0.0151	0.9228 ± 0.0159
GaitScreen, validation-learned convex weighting	0.8743 ± 0.0047	0.8737 ± 0.0045	0.9422 ± 0.0018	0.8423 ± 0.0172	0.9038 ± 0.0227
GaitScreen, Logistic stacking	0.8735 ± 0.0031	0.8724 ± 0.0028	0.9420 ± 0.0016	0.8233 ± 0.0276	0.9195 ± 0.0290

Bio24-LR achieved macro-F1 0.8384 ± 0.0012 and AUROC 0.9315 ± 0.0002, compared with 0.8757 ± 0.0024 and 0.9425 ± 0.0015, respectively, for GaitScreen. The predefined descriptors therefore retained substantial discriminative information, while the full waveform-based model provided gains of 0.0373 macro-F1 and 0.0110 AUROC.

Among the three fusion rules, equal averaging achieved the highest mean macro-F1 (0.8757 ± 0.0024), compared with 0.8737 ± 0.0045 for validation-learned convex weighting and 0.8724 ± 0.0028 for Logistic

stacking. Neither learned alternative improved mean macro-F1 under the adopted protocol; equal averaging was therefore retained as the simpler non-trainable fusion rule.

SUPPLEMENTARY NOTE S9. WALKING-SPEED INFORMATION AND POTENTIAL CONFOUNDING

GaitRec records walking-speed condition categorically as slow, self-selected, or fast and does not provide measured trial-level velocity (Horsak et al., 2020). All trials included in this study were acquired under the self-selected-speed condition. This restriction removes deliberate protocol variation across speed conditions, but it does not make actual walking velocity equal across participants. Pathology-related differences in self-selected speed may therefore contribute to the observed discrimination. Because continuous velocity and a matched speed-controlled cohort are unavailable, a valid speed-adjusted analysis cannot be performed retrospectively. The current results should be interpreted as discrimination under the self-selected-speed GaitRec protocol, not as evidence that GaitScreen identifies pathology independently of walking speed.

SUPPLEMENTARY NOTE S10. COMPUTATIONAL EFFICIENCY BENCHMARK

The computational benchmark used already acquired, segmented, and time-normalised 3×101 GRF trials. On an Apple M4 CPU, single-sample latency was measured for the complete GaitScreen hybrid inference path using batch size 1, 50 warm-up passes, and 1000 timed repetitions with gradient computation disabled. Each measurement included prediction by the multi-scale temporal backbone, CNN, and raw-waveform Logistic Regression components, pathological-class probability conversion, equal averaging, and thresholding. Input preparation and model initialisation were completed before timing. Throughput was measured separately using batches of 64 over 100 repetitions after five warm-up batches.

Computational complexity was compared only among models evaluated under the same CPU benchmark. The full hybrid GaitScreen model contained 285,832 fitted parameters: 143,062 in the multi-scale temporal backbone, 142,466 in the CNN, and 304 fitted coefficients and intercept terms in the binary raw-waveform Logistic Regression. The combined serialised size of the three fitted components was 1.122 MB. GaitScreen was therefore more complex than the raw-waveform Logistic Regression and several compact neural comparators, but smaller by parameter count and serialised size than the evaluated TCN, ResNet1D, and InceptionTime models. These measurements apply to the models evaluated under the present benchmark and are not numerical complexity claims about the originally published systems, whose hardware, preprocessing, model specifications, and timing protocols differ.

Together, the compact serialised size, low single-sample latency, and measured batch throughput support computational scalability for standardised, preprocessed GRF trials. Acquisition throughput and end-to-end clinical workflow were outside the scope of this benchmark.

SUPPLEMENTARY NOTE S11. RELATION TO PRIOR GRF CLASSIFICATION STUDIES

Representative GRF studies have investigated healthy-versus-pathological and multiclass gait classification using parameter- and PCA-based representations, convolutional networks, boosted classifiers, explainable-learning pipelines, and Transformer-style temporal models (Slijepcevic et al., 2018; Pandey et al., 2022; Jani et al., 2022; Mulwa et al., 2024; Wang et al., 2025). Supplementary Table S9 compares their inputs, model families, tasks, evaluation designs, and decision-level reporting. Differences in cohorts, class definitions,

preprocessing procedures, and validation protocols limit direct numerical comparison with GaitScreen. The literature-inspired comparators were adapted from these model families and evaluated under the retained GaitRec cohort; they are not exact reproductions of the published systems or performance values.

SUPPLEMENTARY TABLE S9. RELATION TO PRIOR GRF CLASSIFICATION STUDIES

Table S9. Position of the present analysis within representative GRF-classification literature. Published results are not compared numerically because the studies differ in cohort construction, class definitions, inputs, preprocessing, and validation design. “Not a stated focus” means that decision-oriented operating-threshold analysis was not identified as a primary aim in the cited source.

Slijepcevic et al. (2018)	Pandey et al. (2022)	Jani et al. (2022)	Mulwa et al. (2024)	Wang et al. (2025)	Present study
**GRF representation/input**					
GRF parameters and PCA-based representations	One-dimensional GaitRec GRF input	Processed bilateral GaitRec GRF/CoP signals with selected metadata	GaitRec input for an MLP; HugaDB input for a Random Forest	GaitRec GRF trajectories	Three GRF channels, each with 101 stance samples; 303-dimensional flattened input for Logistic Regression
**Model family**					
Classical classifiers	1D CNN with SVM, KNN, and Naive Bayes comparators	Boosted tree classifiers, including optimised CatBoost	Supervised classifiers with GMM-LIME	Transformer with attention-based feature extraction	Multi-scale temporal backbone, CNN, and raw-waveform Logistic Regression with equal averaging
**Main task/output**					
Healthy-versus-impaired and five-class functional-gait classification	Binary healthy-versus-gait-disorder classification	Detailed multiclass anatomical abnormality classification	Classification plus stability of local explanations	Five-class healthy, hip, knee, ankle, and calcaneus classification	Trial-level healthy-versus-pathological review-prioritisation score
**Validation/evaluation design**					
Study-specific binary and multiclass evaluation in 440 participants	Fivefold cross-validation over randomly divided samples	Study-specific train/test evaluation on held-out samples	Test-set performance and repeated explanation-stability analyses	Cross-validation experiments	One fixed subject-exclusive train/validation/test partition; ten matched-seed refits
**Decision-level or threshold reporting**					
Classification performance; prescreening thresholds were not a stated focus	Accuracy, precision, recall, and F1; validation-selected review thresholds were not reported	Classification metrics; review-threshold analysis was not a stated focus	Accuracy, AUC, and explanation-similarity measures; referral thresholds were not a stated focus	Classification accuracy and attention-based interpretation; prescreening thresholds were not a stated focus	Validation-selected thresholds; sensitivity, specificity, FNR/FPR, pathology-family behaviour, and post-hoc subject aggregation/calibration
**Relevance to the present study**					
Reports early parameter- and PCA-based GRF comparisons	Provides a convolutional GRF-classification reference	Illustrates feature-rich ensemble classification on GaitRec	Highlights explanation stability and the need to match explanations to the prediction model	Represents a recent attention-based temporal model	Integrates compact modelling, common benchmarking, and decision-oriented evaluation

SUPPLEMENTARY NOTE S12. DATASET SELECTION AND REPRESENTATIVE PUBLIC ALTERNATIVES

GaitRec was selected for the present analysis because it combines a comparatively large public clinical cohort with standardised bilateral force-plate recordings, vertical, anterior–posterior, and mediolateral stance-phase GRF trajectories, subject and trial identifiers, and detailed lower-limb orthopaedic pathology and limb-level metadata (Horsak et al., 2020). These properties support a subject-exclusive healthy-control-versus-pathological-gait evaluation while retaining substantial heterogeneity across lower-limb impairment families.

Other public gait datasets are available. Supplementary Table S10 compares four representative resources verified from their official repositories or original data publications. The two PhysioNet datasets provide foot-sensor force signals from neurological cohorts, whereas Daphnet and mPower provide wearable or smartphone inertial recordings (Hausdorff, 2008, 2000; Roggen et al., 2010; Bächlin et al., 2010; Bot et al., 2016). Their target populations, signal definitions, acquisition protocols, repeated-measure structures, and labels differ from those used in GaitRec. These differences do not preclude future external evaluation, but they require harmonisation of the sensor representation, preprocessing, target labels, and eligible population. The present results constitute internal validation within GaitRec and do not establish cross-dataset generalisability.

SUPPLEMENTARY TABLE S10. COMPARISON WITH REPRESENTATIVE PUBLIC GAIT DATASETS

Table S10. Comparison of GaitRec with representative publicly available gait datasets relevant to pathological-gait analysis. "Public, controlled access" indicates that qualified researchers may request access subject to the repository's data-use requirements.

**GaitRec** (Horsak et al., 2020)	**Gait in Parkinson's Disease** (Hausdorff, 2008)	**Gait in Neurodegenerative Disease** (Hausdorff, 2000)	**Daphnet Freezing of Gait** (Roggen et al., 2010; Bächlin et al., 2010)	**mPower** (Bot et al., 2016)
**Population/pathology focus**				
Healthy controls and heterogeneous lower-limb musculoskeletal impairments	93 participants with idiopathic Parkinson's disease and 73 healthy controls	Parkinson's disease ($n = 15$), Huntington's disease ($n = 20$), amyotrophic lateral sclerosis ($n = 13$), and healthy controls ($n = 16$)	Ten participants with Parkinson's disease who performed laboratory walking tasks; freezing occurred in eight	Large longitudinal smartphone study including participants with and without Parkinson's disease; public, controlled access
**Signal/sensing modality**				
Standardised laboratory force plates; bilateral stance-phase trials	Eight force sensors beneath each foot during approximately two minutes of walking	Force-sensitive resistors beneath the left and right feet	Three triaxial accelerometers on the ankle, thigh, and trunk	iPhone accelerometer and gyroscope during walking and balance tasks
**GRF dimensionality or relevant input**				
Vertical, anterior–posterior, and mediolateral GRF; processed trajectories contain 101 stance samples	Vertical underfoot force from 16 sensors plus summed left/right signals; no anterior–posterior or mediolateral GRF	Left/right force-proportional signals and derived stride-time measures; no anterior–posterior or mediolateral GRF	Nine acceleration channels; no GRF	Triaxial inertial recordings; no GRF
**Subject identifiers/repeated trials**				
Explicit subject, session, and trial identifiers; repeated trials and sessions	Subject-coded records; multiple recordings or conditions are available for some participants	One subject-coded record per participant, with paired left/right signals	Subject- and recording-coded observations across several walking tasks	Participant-linked repeated remote assessments
**Label structure**				
Healthy control; hip, knee, ankle, and calcaneus families with detailed subclasses	Parkinson's disease versus healthy control, with disease-severity and study metadata	Four neurological/control groups with disease-specific severity or duration metadata	Samples labelled outside experiment, no freezing, or freezing of gait	Parkinson's disease/control status, medication timing, and task metadata
**Relevance to the present study**				
Selected because scale, three-component GRF, identifiers, and orthopaedic metadata jointly match the present subject-exclusive task	A relevant force-based neurological resource, but the population, sensor geometry, force components, and protocol differ from GaitRec	Supports neurological gait analysis, but is smaller and uses in-shoe force-proportional signals and different disease labels	Useful for event-level freezing detection, but differs in modality, population, and target definition from healthy-versus-pathological GRF classification	Valuable for remote neurological assessment, but its sensing, acquisition environment, and labels are not interchangeable with laboratory three-component orthopaedic GRF

REFERENCES

Bächlin, M., Plotnik, M., Roggen, D., Maidan, I., Hausdorff, J. M., Giladi, N., et al. (2010). Wearable assistant for Parkinson's disease patients with the freezing of gait symptom. *IEEE Transactions on Information Technology in Biomedicine* 14, 436–446. doi:10.1109/TITB.2009.2036165

Bot, B. M., Suver, C., Chaibub Neto, E., Kellen, M., Klein, A., Bare, C., et al. (2016). The mPower study, Parkinson disease mobile data collected using ResearchKit. *Scientific Data* 3, 160011. doi:10.1038/sdata.2016.11

[Dataset] Hausdorff, J. M. (2000). Gait in neurodegenerative disease database. doi:10.13026/C27G6C

[Dataset] Hausdorff, J. M. (2008). Gait in Parkinson's disease. doi:10.13026/C24H3N

Horsak, B., Slijepcevic, D., Raberger, A.-M., Schwab, C., Worisch, M., and Zeppelzauer, M. (2020). GaitRec, a large-scale ground reaction force dataset of healthy and impaired gait. *Scientific Data* 7, 143. doi:10.1038/s41597-020-0481-z

Jani, D., Varadarajan, V., Parmar, R., Bohara, M. H., Garg, D., Ganatra, A., et al. (2022). An efficient gait abnormality detection method based on classification. *Journal of Sensor and Actuator Networks* 11, 31. doi:10.3390/jsan11030031

Mulwa, M. M., Mwangi, R. W., and Mindila, A. (2024). GMM-LIME explainable machine learning model for interpreting sensor-based human gait. *Engineering Reports* 6. doi:10.1002/eng2.12864

Pandey, C., Roy, D. S., Poonia, R. C., Altameem, A., Nayak, S. R., Verma, A., et al. (2022). GaitRec-Net: A deep neural network for gait disorder detection using ground reaction force. *PPAR Research* 2022, 1–10. doi:10.1155/2022/9355015

[Dataset] Roggen, D., Plotnik, M., and Hausdorff, J. M. (2010). Daphnet freezing of gait. doi:10.24432/C56K78

Slijepcevic, D., Zeppelzauer, M., Gorgas, A.-M., Schwab, C., Schuller, M., Baca, A., et al. (2018). Automatic classification of functional gait disorders. *IEEE Journal of Biomedical and Health Informatics* 22, 1653–1661. doi:10.1109/JBHI.2017.2785682

Wang, J., Son, N., Kim, S., and Shin, M. (2025). GRF-Former: Advanced transformer-based model for multi-class gait disorder classification using ground reaction force data. *SSRN Electronic Journal* doi:10.2139/ssrn.5508918
